# Diffuse cardiac lymphatic involvement by metastatic neuroendocrine carcinoma mimicking hypertrophic cardiomyopathy: a case report

**DOI:** 10.1186/1757-1626-2-9127

**Published:** 2009-12-02

**Authors:** Takeshi Kondo, Riko Kitazawa, Emiko Kawata, Kiyoshi Mori, Sohei Kitazawa

**Affiliations:** 1 Division of Pathology (Diagnostic Molecular Pathology Unit), Kobe University Graduate School of Medicine, 7-5-1 Kusunoki-cho, Chuo-ku, Kobe 650-0017, Japan

## Abstract

We describe an autopsy case of non-functioning pancreatic neuroendocrine carcinoma metastasizing to the myocardium. A 63-year-old Japanese man was admitted to the hospital presenting with dyspnea. Echocardiography revealed marked left ventricular hypertrophy and diffuse myocardial thickening with pericardial effusion. The patient died of heart failure. An autopsy revealed that the whole pancreas, weighing 400 g, was occupied by tumor cells with neuroendocrine differentiation. The heart, weighing 780 g, showed numerous metastatic nodules and diffuse myocardial thickening. Histopathologically, the tumor was diagnosed as non-functioning pancreatic neuroendocrine carcinoma. Immunohistochemical analysis for D2-40 disclosed severe lymphatic infiltration of tumor cells, characterized by diffuse thickening of the myocardium.

## Introduction

Pancreatic neuroendocrine tumors are rare neoplasms accounting for less than 5% of all primary pancreatic malignancies [1]. Non-functioning pancreatic neuroendocrine tumors demonstrate endocrine differentiation but lack a clinical syndrome of hormone hypersecretion. Presentation is related to the mass effect of the tumor, and patients can present with advanced metastatic disease but relatively few symptoms [1]. Here, an autopsy case of non-functioning pancreatic neuroendocrine carcinoma metastasizing to the myocardium is reported.

## Case presentation

A 63-year-old Japanese man was admitted to our hospital complaining of dyspnea. He had a history of smoking (30 cigarettes a day for 40 years) and had been diagnosed as hypertensive in the past. Echocardiography revealed marked left ventricular hypertrophy and diffuse myocardial thickening with pericardial effusion, simulating hypertrophic cardiomyopathy. Computed tomography (CT) revealed multiple lymphadenosis of the superior mediastinum, the neck and the hilum of the lung. Biopsies of cervical lymph nodes and the pericardium revealed metastatic neuroendocrine tumor suggestive of pancreatic, digestive tract, or lung origin. A cytologic examination from pericardial effusion was negative for malignancy. Although the primary site was not identified, the tumor was diagnosed as metastatic atypical carcinoid. After two courses of anticancer chemotherapy (carboplatin, irinotecan), the patient underwent radiation therapy. In spite of the intensive treatment, severe heart failure and anemia developed gradually. The patient died of heart failure, and an autopsy was conducted to disclose the origin of the cancer and the nature of the systemic disease, including cardiac muscle lesions.

## Pathologic Findings

**Figure 1:**
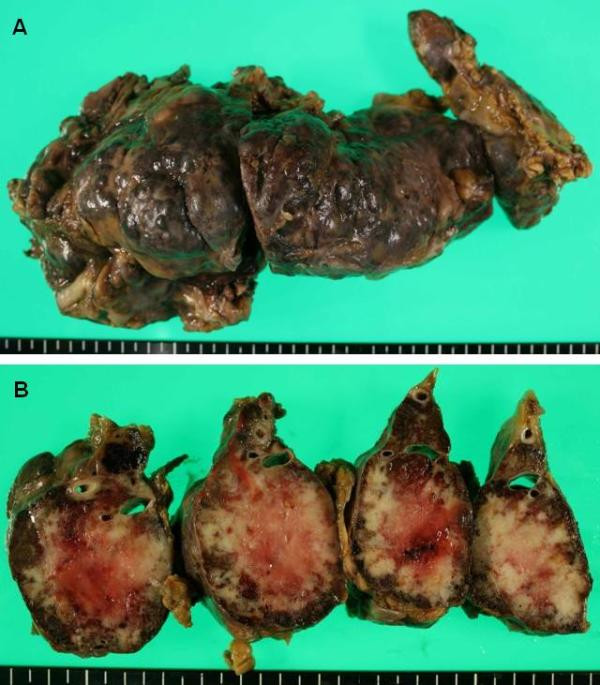
**Macroscopic findings of the pancreas**. A: pancreas, weighing 400 g, was occupied by the tumor. B: on the cut surface, residual pancreatic tissue was not macroscopically identifiable.

**Figure 2:**
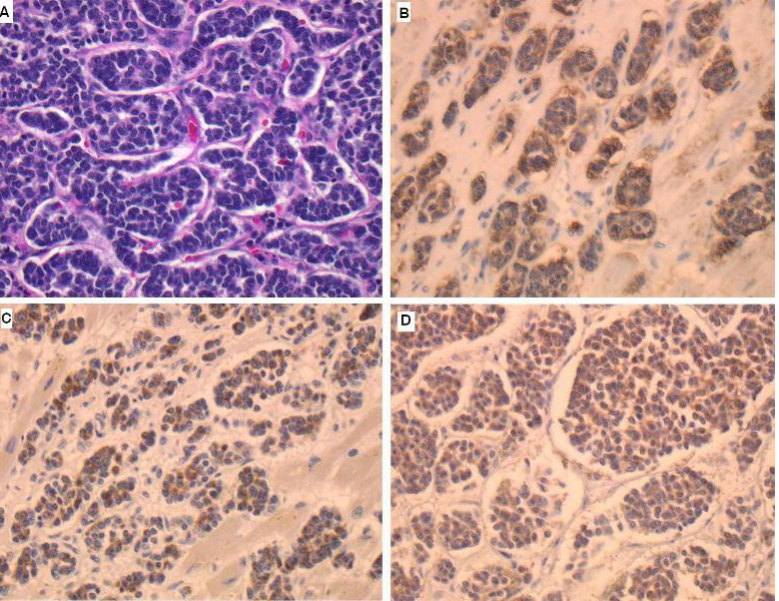
**Microscopic findings of the tumor**. A: the tumor cells showed nested or trabecular architecture suggesting neuroendocrine differentiation. B, C and D: tumor cells were positive for neuroendocrine markers (synaptophysin (B), NCAM (CD56) (C), and chromogranin A (D)).

**Figure 3:**
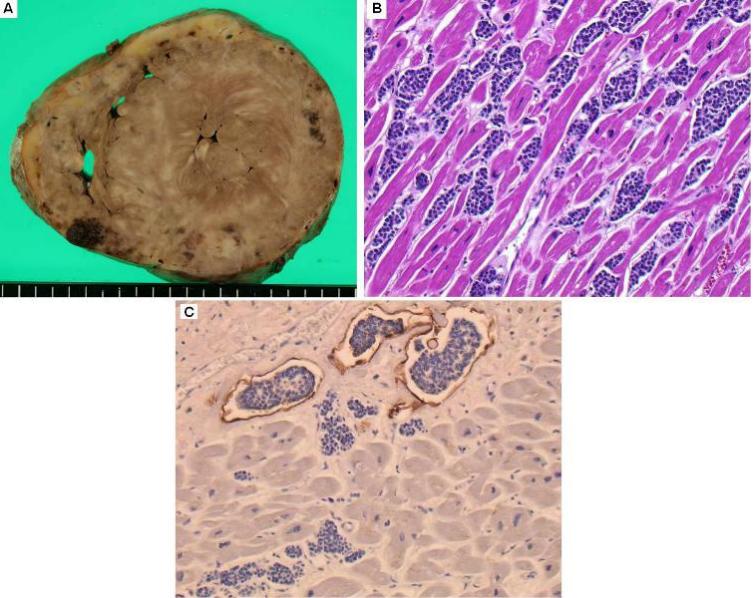
**Macroscopic and microscopic findings of the heart**. A: cut surface of the heart. The heart weighed 780 g and showed numerous metastatic nodules and diffuse myocardial thickening, simulating hypertrophic cardiomyopathy. B: metastatic tumor cells invaded the myocardium with stromal edema (HE). C: immunohistochemical analysis for D2-40 demonstrated severe lymphatic infiltration of tumor cells.

Grossly, the whole pancreas, weighing 400 g, was occupied by a tumor (Fig. 1AB) of cells with a characteristic nested or trabecular growth pattern suggesting neuroendocrine differentiation (Fig. 2A). Immunohistochemically, the tumor cells were positive for major neuroendocrine markers, such as synaptophysin, NCAM (CD56), and chromogranin A (Fig. 2BCD). On the other hand, the heart weighed 780 g and showed numerous metastatic nodules and diffuse myocardial thickening (Fig. 3A). Metastatic tumor cells invaded the myocardium with stromal edema (Fig. 3B). Moreover, immunohistochemical analysis for D2-40 demonstrated severe lymphatic infiltration of tumor cells (Fig. 3C). Aggressive metastasis was confirmed at various loci including the liver, lungs, pleura, kidneys, spleen, adrenal glands, vertebrae, ribs, and lymph nodes. Based on the above findings, the tumor was diagnosed as primary pancreatic neuroendocrine carcinoma with systemic metastases.

## Discussion

Non-functioning pancreatic endocrine carcinomas are hormonally silent by definition. Growing large, they display symptoms attributable to local disease or distant metastases. In autopsy series, the heart is a relatively common site for metastatic tumors, which are at least 100 times more common than primary malignant cardiac tumors [2,3]. The incidence of cardiac metastases is on the rise due to longer patient survival attendant upon improved chemotherapy and radiotherapy, coupled with increasingly sensitive diagnostic modalities. Although difficult to define, the incidence of cardiac involvement in patients with malignant neoplasms is probably above 10% [4]. Metastases reach the heart and the pericardium through retrograde lymphatic spread, the bloodstream, direct invasion, or by direct venous extension. In our present case, myocardial metastases reflected severe lymphatic invasion of tumor cells. Myocardial metastases directly from neuroendocrine carcinoma are rare, with a reported incidence of 2% to 4%, and usually found in patients with widespread metastatic disease and extensive liver infiltration [5,6]. Interestingly, a similar case of myocardial metastasis from pancreatic carcinoma (specific histology unknown) presenting as acute myocardial infarction was reported [7]. Myocardial metastases may cause an unexpected cardiac manifestation.

In conclusion, a rare autopsy case of non-functioning pancreatic neuroendocrine carcinoma metastasizing to myocardium and mimicking hypertrophic cardiomyopathy is reported.

## Consent

Written informed consent was obtained from the patient for the publication of this case report with accompanying images. A copy of the written consent is available for reviewing by the Editor-in-Chief of the journal.

## Competing interests

The authors declare that they have no competing interests.

## Authors' contributions

All authors analyzed and interpreted the autopsy data. RK and SK performed autopsy and histological examinations. EK and KM assisted with the histological examinations. TK is the major contributor to the writing of the manuscript. SK supported the case analysis and the writing of the manuscript. All authors read and approved the final manuscript.
